# Supplementary material for: Patterns of Intron Gain and Loss in Fungi
Source: PLoS Biol. 2004 Nov 30;2(12):e422. doi: 10.1371/journal.pbio.0020422 (PMC532390; doi:10.1371/journal.pbio.0020422)
Supplement: Table S1 — Also available at http://genes.mit.edu/NielsenEtAl/. (4.3 MB ZIP). [file pbio.0020422.st001.zip › NielsenEtAl/html/1231.html]

AN5626.1.NCU06836.1.MG03201.1.FG00330.1


```
 CLUSTAL W (1.82) Multiple Sequence Alignments - Introns Inserted


Sequence 1: NCU06836.1	631 aa
Sequence 2: FG00330.1	683 aa
Sequence 3: AN5626.1	670 aa
Sequence 4: MG03201.1	635 aa
Alignment Length: 685 aa
Number Identitical Residues: 439 aa
Alignment Score (without introns) 20009


MG03201.1 	--------------~-------------------------------~-----MLDN1LEE
NCU06836.1	--------------~-------------------------------~------MSD1FEE
FG00330.1 	MASPAPAPAPAAAE~TVSLKSEETQAPAKETKGPVVAEAHLVDTYQ1HPHRPHLAN1LEE
AN5626.1  	-MSDGPIAPPKPAV0VAEAHEVDTFHVPKAFFD------------K~HPSGPHLKN~LDE
          	  : .. ... .:    .  . .:   ..   .            .  .  . : : ::*

MG03201.1 	YQQLYKESISEPKQFWARMARELLTWSRDFETVYSGSLSAGDSAWFLEGELNASYNCIDR
NCU06836.1	YQKLYKQSITEPNVFWAERARELLSWSKDFQTVHSGSLANGDNAWFVEGELNASYNCVDR
FG00330.1 	YKRLYKESITQPEKFWAERARELISWDNDFKTTQSGSLLNADVSYFNEGRLNASYNCVDR
AN5626.1  	YKKLYEESIRSPDVFWARKARELLTFDKDFQTTRIGSLENGDVAWFPEGRLNASFNCVDR
          	*::**::** .*. ***. ****:::..**:*.  ***  .* ::* **.****:**:**

MG03201.1 	HALKDPNRVAIIYEADDPSSGRTLTYGELMREVCRTAHVLRQMGVRKGDTVAVYLPMIPE
NCU06836.1	HAHKDPNRVAIIYEADEPGQGRNVTYGELLREVSKLAHVLTNLGVRKGDTVAIYLPMIPE
FG00330.1 	HAFKDPDRVAIIYEADEPSEGRNVTYGELLREVSRTAWVLKQMGVRKGDTVAIYLPMIPE
AN5626.1  	HAIKNPNKVAIIYEADEPNEGRTITYGELLREVSRVAWVLKQRGVKKGDTVAIYLPMIPE
          	** *:*::********:*..**.:*****:***.: * ** : **:******:*******

MG03201.1 	ALIAFLAVTRIGAVHSVVFAGFSADSLRDRVVDGQSKVVITTDEGKRGGKLIGTKRIVDE
NCU06836.1	AIVAMLACTRIGAIHSVVFAGFSSDSLRDRVVDAQSKVVITTDEGKRGGKLIGTKKIVDE
FG00330.1 	AIIALMACVRIGAVHSVIFAGFSSDSLRDRVVDAGSKVVITTDEGKRGGKLIGTKKIVDD
AN5626.1  	AIIAFLACSRIGAVHSVVFAGFSSDSLRDRVLDAGSKVVITTDEGKRGGKVIGTKRIVDE
          	*::*::*  ****:***:*****:*******:*. ***************:****:***:

MG03201.1 	ALKNCPDVSHVLVYKRTGADVPMMQGRDFWWHEEVEKWPNYIPPVPMNSEDPLFLLYTSG
NCU06836.1	ALQQCPDVRNVLVYKRTGADISMTPGRDLWWHEEVEKYPAYYTPVAMASEDPLFLLYTSG
FG00330.1 	ALKQCPAVENVLVYKRTGSEIPWTSGRDFWWHEEVEKWPNYFPPEPVASEDPLFLLYTSG
AN5626.1  	GLKQCPDVSTVLVYKRTGAEVPWTEGRDIWWHEEVEKYPAYIAPDSVNSEDPLFLLYTSG
          	.*::** *  ********:::.   ***:********:* * .* .: ************

MG03201.1 	STGKPKGVMHTTAGYLLGAAMTGKYVFDIHDGDRFFCGGDVGWITGHTYVVYAPLLLGVS
NCU06836.1	STGKPKGVAHSTGGYLLGAAMTGKYVFDIHDGDRYFCGGDVGWITGHTYVLYAPLLLGVS
FG00330.1 	STGKPKGVLHTTAGYLLGAAMTGKYVFDIHEGDRYFCGGDVGWITGHTYVVYAPLMLGVS
AN5626.1  	STGKPKGVMHTTAGYLLGAAMTGKYVFDIHDDDRYFCGGDVGWITGHTYVVYAPLLLGCS
          	******** *:*.*****************:.**:***************:****:** *

MG03201.1 	TVVFEGTPAYPTFSRYWDIIDQHKITHFYVAPTALRLLKRAGDQWVKHEMKHLRVLGSVG
NCU06836.1	TVVFEGTPAYPNFSRYWDIIEEHKVTQFYVAPTALRLLKRAGDHHVRNEMKHLRVLGSVG
FG00330.1 	TVVFEGTPAYPNFSRYWDIIEKHDVTQFYVAPTALRLLKRAGDEHVRGKFAHLRVLGSVG
AN5626.1  	TVVFESTPAYPDFSRYWDVIEKHKVTQFYVAPTALRLLKRAGDHHIHHKMEHLRVLGSVG
          	*****.***** ******:*::*.:*:****************. :: :: *********

MG03201.1 	EPIAAEVWKWYFEIVGKEQAQIVD~TYWQTETGSNVICPLAGVTPTKPGSASLPFFGIEP
NCU06836.1	EPIAAEVWKWYYDVVGKGRAQICD~TYWQTETGSNVITPLAGVTPTKPGSASFPFFGIEP
FG00330.1 	EPIAAEIWKWYFEVIGKEECHIVD0TYWQTESGSHVITPLAGITPTKPGSCSLPFFGVEP
AN5626.1  	EPIAAEVWKWYFEKVGKEEAHICD0TYWQTETGSNVITPLAGVTPTKPGSASLPFFGIEP
          	******:****:: :** ..:* * ******:**:** ****:*******.*:****:**

MG03201.1 	AIIDPVSGEEIHGNDVEGVLGFKQAWPSMARTVWGAHKRYMETYMNVYNGFY0FTGDGAA
NCU06836.1	ALVDPVTGEEIHGNDVEGVLAFKQPWPSMARTVWGAHKRYMETYLHVYKGYY~FTGDGAA
FG00330.1 	ALIDPVSGEEIHGNDVEGVLTFKQSWPSMARTVWGAHNRYKETYLDVYKGYY~FTGDGAA
AN5626.1  	AIIDPVSGEEISGNDVEGVLAFKQPWPSMARTVWGAHKRYMDTYLNVYKGYY0FTGDGAG
          	*::***:**** ******** ***.************:** :**:.**:*:* ******.

MG03201.1 	RDHEGFYWIRGRVDDVVNVSGHRLSTAEIEAALLEHH~YVAEAAVVGIQDELTGQAVNAF
NCU06836.1	RDHEGFYWIRGRVDDVVNVSGHRLSTAEIEAALIEHH~SIAEAAVVGVADELTGQAVNAF
FG00330.1 	RDHEGFYWIRGRVDDVVNVSGHRLSTAEIEAALLEHH~AVADAAVVGIADELTGQAVNAF
AN5626.1  	RDHEGYYWIRGRVDDVVNVSGHRLSTAEIEAALIEHP1MVAEAAVVGIADELTGQAVNAF
          	*****:***************************:**   :*:*****: ***********

MG03201.1 	VSLAEHVTTDNDESLRKELITQVRRSIGPFAAPKAVFVVPDLPRTRSGKIMRRILRKILA
NCU06836.1	VAVKEGTQIN--DALRKEFVLQVRRSIGPFAAPKAIYIVPDLPKTRSGKIMRRILRKIVA
FG00330.1 	VDLEKNVEAT--DALRKELIVQVRKSIGPFAAPKAVYIVPDMPKTRSGKIMRRVLRKIVA
AN5626.1  	VSLKEGNETN--DQVRKDLILQVRKSIGPFAAPKAVFVVDDLPKTRSGKIMRRILRKILS
          	* : :       : :**::: ***:**********:::* *:*:*********:****::

MG03201.1 	GEEDQLGDVST0LSDPTVVDRIITTVHEYKKK
NCU06836.1	GEEDQLGDITT0LSDPSVVAKIIDVVHAQRQ-
FG00330.1 	GEEDQLGDTTT0LSDPSVVDKIIKAVHDAKRK
AN5626.1  	GEEDSLGDIST0LSDPSVVERIIATVHASRGK
          	****.*** :* ****:** :** .**  : .
```
